# Using generalizability theory to evaluate the comparative reliability of developmental measures in neurogenetic syndrome and low-risk populations

**DOI:** 10.1186/s11689-020-09318-1

**Published:** 2020-06-05

**Authors:** Lisa R. Hamrick, Alison M. Haney, Bridgette L. Kelleher, Sean P. Lane

**Affiliations:** Department of Psychological Sciences, Purdue University, 703 Third Street, West Lafayette, IN 47907 USA

**Keywords:** Angelman, Communication and Symbolic Behavior Scales, Generalizability theory, Neurogenetic, Prader-Willi, Reliability, Social communication, Williams

## Abstract

**Background:**

The lack of available measures that can reliably characterize early developmental skills in children with neurogenetic syndromes (NGS) poses a significant challenge for research on early development in these populations. Although syndrome-specific measures may sometimes be necessary, a more cost- and time-efficient solution would be to identify existing measures that are appropriate for use in special populations or optimize existing measures to be used in these groups. Reliability is an important metric of psychometric rigor to consider when auditing and optimizing assessment tools for NGS. In this study, we use Generalizability Theory, an extension of classical test theory, as a novel approach for more comprehensively characterizing the reliability of existing measures and making decisions about their use in the field of NGS research.

**Methods:**

We conducted generalizability analyses on a popular early social communication screener, the Communication and Symbolic Behavior Scales—Infant-Toddler Checklist (CSBS-ITC), collected on 172 children (41 Angelman syndrome, 30 Prader-Willi syndrome, 42 Williams syndrome, 59 low-risk controls).

**Results:**

Overall, the CSBS-ITC demonstrated at least adequate reliability in the NGS groups included in this study, particularly for the Prader-Willi and Williams syndrome groups. However, the sources of systematic error variance in the CSBS-ITC varied greatly between the low-risk control and NGS groups. Moreover, as unassessed in previous research, the CSBS-ITC demonstrated substantial differences in variance sources among the NGS groups. Reliability of CSBS-ITC scores was highest when averaging across all measurement points for a given child and was generally similar or better in the NGS groups compared to the low-risk control group.

**Conclusions:**

Our findings suggest that the CSBS-ITC communicates different information about the reliability of stability versus change, in low-risk control and NGS samples, respectively, and that psychometric approaches like Generalizability Theory can provide more complete information about the reliability of existing measures and inform decisions about how measures are used in research on early development in NGS.

## Background

Children with neurogenetic syndromes (NGS) demonstrate severe developmental delays that span multiple domains. Reliably operationalizing and measuring these delays is important for a number of reasons, including determining areas of need and monitoring response to intervention. While many of the most commonly used developmental measures function reliably in typically developing (TD) populations, a current challenge in NGS is determining whether these measures are similarly reliable when applied with children with severe and multidimensional delays. Importantly, measures with low reliability are more likely to either miss effects that are truly present (i.e., type II errors) or suggest the presence of statistically significant effects that are due to error (i.e., type I errors). These risks are particularly hazardous in NGS research, which often deals with very small, highly heterogeneous samples, and whose findings are often given substantial weight in determining the effectiveness of treatment protocols, especially in clinical trials [1]. Thus, there is a need for comprehensive psychometric evaluation of measurement tools in NGS. That is, can a measure be used reliably in its current form, does it require optimization for use in NGS, or is wholesale replacement by an instrument specifically designed to measure developmental skills in NGS justified? This critical evaluation of the reliability of existing measures in NGS is one aspect of this comprehensive evaluation that will provide better understanding of the strengths and limitations of common tools in the field.

In this study, we present generalizability theory (GT) as an underutilized and parsimonious method for evaluating the reliability of measures used in NGS research. GT is an extension of classical test theory [2] that allows for the evaluation of the effects of multiple sources of variance, and their interactions, on the reliability of a measure. We demonstrate the advantages of using GT by examining the variance decomposition and reliability of a popular early social communication screening measure, the Communication and Symbolic Behavior Scales–Infant Toddler Checklist (CSBS-ITC [3];), in three NGS populations and a low-risk comparison group.

### Threats to reliability in NGS

Classical reliability is the ability to measure a certain phenomenon consistently, such that similar results should be obtained across repeated evaluations when the phenomenon of interest is stable. Any variation in scores across evaluations is attributed to error, which can occur randomly or systematically. While random error is difficult or impossible to avoid, systematic error, when identified, can be reduced by adjusting certain characteristics of the measure. Importantly, assessment (e.g., longitudinal) and methodological (e.g., GT) approaches can be used to parse sources of systematic and random measurement variability into random effects that inform how a given measure’s reliability can be optimized. Using measures with low reliability (i.e., large error) increases the chances of error in statistical hypothesis testing. Thus, the goal in developing any measure is to reduce the overall amount of error, which in turn will improve the measure’s reliability.

Several overarching attributes common to NGS groups increase the risk of measurement unreliability. First, a vast amount of heterogeneity in phenotypic presentation exists within most syndrome populations. Thus, even individuals with the same genetic diagnosis demonstrate immense variability across developmental domains, which may not necessarily be the target of measurement, but that may nevertheless differentially contribute to error. Second, early development in children with NGS is often severely delayed compared to that of similarly aged TD children who serve as the normative sample for most developmental measures. As such, many measures focus on skills that are more advanced than what would be expected for a child with NGS, which can lead to many children with NGS receiving scores that cluster around the measure floor. Relatedly, children with NGS may receive very similar scores across multiple measurement points over time due to exhibiting a slower rate of development than would be expected in TD populations. Combined, these factors contribute to many commonly used assessment tools lacking enough granularity to capture meaningful change in skills over time for children with NGS [4, 5]. A lack of between- and within-person variability in scores limits the ability of researchers and clinicians to accurately estimate true skills, separate from random variation due to error, among children with NGS.

A major question in the field, then, is how to move forward with assessing early development in NGS given the limitations of current measurement options. One approach has been to move away from using existing standardized measures and instead turn to new measures that have either been tailored to specific syndromic populations or that draw from naturalistic samples of behavior with the ability to capture more variability within individuals (e.g., [6–9]). While sometimes necessary, this approach comes at a cost: validating new measures requires significant time, resources, and expertise, and can also limit the ability to compare with the vast amount of existing literature using more popular measures in typical development.

Another option is to critically evaluate existing measures to determine if and how these tools can be used appropriately in NGS. In fact, an NIH work group focused on identifying appropriate outcome measures for clinical trials in fragile X syndrome recommended an approach of “borrow, adapt, and evaluate,” which involves examining measures being used in other clinical populations for feasibility in fragile X syndrome [10]. By embracing this approach as a first step for evaluating measurement tools in NGS, the field may potentially avoid unnecessary efforts towards collecting and analyzing intricate behavioral samples or developing new measures altogether. However, a first step to the “borrow, adapt, and evaluate” approach will be to assess the psychometric properties of existing measures in NGS populations, including one of the most commonly discussed metrics of psychometric rigor: reliability.

### Generalizability theory

Reliability is the ability to measure a certain phenomenon consistently such that similar results should be obtained across repeated evaluations. Reliability is commonly measured using classical test theory methods, including internal consistency, inter-rater reliability, and test-retest reliability, all of which focus on the stability of point estimates at a single measurement point or across pairs of measurement points. However, these common metrics of reliability do not inform the reliability of measurements across expected periods of change—such as natural development or acute response to intervention. That is, classical test theory methods do not answer the question of whether change over time as measured by a particular instrument is considered true change or measurement error, and what sources of variance may be contributing to changes in scores over time.

GT is a more flexible statistical framework for evaluating the reliability of a measure [11]. As an expansion of classical test theory, GT acknowledges that variance in an observed score comes from multiple sources [12]. Classical test theory only considers a single source of variance, while GT allows for simultaneous consideration of between-person, within-person, and the remaining variance, which is a combination of unattributed variance and true error variance [13]. While GT can be used to construct various types of reliability estimates, it also provides insight into the sensitivity of a measure by estimating the amount of variance accounted for by individual and group factors.

There are two main components of a GT analysis: the generalizability study or “G Study” and the decision study or “D Study”. The first step (G Study) estimates potential sources of variance in observed scores by computing linear combinations of analysis of variance (ANOVA) mean squares, which are estimates of variance across groups [11]. These analyses provide generalizability estimates, which represent a raw proportion of the total variance accounted for by each included factor. Such factors can be specified to include person, item content, time of measurement, or other variables that are thought to systematically relate to the construct of interest (e.g., a child’s age would be considered when evaluating a developmental measure). The output of GT analyses is random effect variance components, which are commonly expressed as percentages (out of 100%), that describe the relative contribution of each factor to the total variance. For example, if the variance due to person is 12%, that means that 12% of the total variance is due to individual differences not accounted for by other included factors.

In the second step of GT (D Study), variance components are used to estimate various forms of reliability [14]. For longitudinal designs, four reliability estimates are typically computed using the procedure outlined by Cranford et al. [14]. *R*_*KF*_ represents the reliability of the average scale ratings from all items and all measurement periods. If a researcher gave participants the same measure every day for 1 week, *R*_*KF*_ would be the between-person reliability when the average was taken across all 7 days for each participant. *R*_*1F*_ represents the reliability of one fixed measurement point (e.g., when collected on the same day or at the same age). In a repeated-measures study, *R*_*1F*_ would be the reliability if the researcher selected one single date from a week-long data collection period and compared all participants on that single day, perhaps to control for effects of day of the week. In developmental research, studies often collect measures at specified ages (e.g., all participants are assessed when they are 6, 9, and 12 months old). In this case, *R*_*1F*_ would be the reliability when comparing all participants of the same age. *R*_*1R*_ represents the reliability of one randomly selected measurement point (e.g., measurement points that may be collected on different days or at different ages), as is commonly the case in developmental research. In this way, *R*_*1R*_ demonstrates how the level of a given measure can be impacted both by differences at measurement points and how participants may respond uniquely at that measurement point [13]. Finally, *R*_*C*_ represents the reliability of change for a given measure and is particularly relevant to developmental research. Different from test-retest reliability, *R*_*C*_ considers the proportion of variability that is due to systematic changes over time within individuals. High *R*_*C*_ indicates that a measure can reliably detect variation in scores for the same person across time, whereas low *R*_*C*_ may indicate a lack of reliable variation in scores over and above the common effect of time across all persons.

GT can address specific measurement challenges faced by early developmental research in NGS. First, GT allows researchers to characterize specific sources of measurement error variance inherent in NGS groups. For example, it is possible to determine how much variability stems from individual differences, item content or wording, and developmental trajectories. Importantly, GT also allows researchers to examine how *interactions* across these factors contribute to variance. For example, person-level differences and item content may interact, suggesting that certain individuals tend to systematically score higher (or lower) on certain items of the measure compared to others. This pattern may indicate that certain items are not capturing the intended construct for a certain group of individuals due to a secondary variable such as speech or motor ability.

Second, GT demonstrates how different sources of variance affect the reliability of measurement tools when they are applied to NGS samples but were designed for use in TD populations. The GT reliability estimates can indicate whether a measure can reliably differentiate children within a certain syndromic population, as well as whether the measure can reliably detect within-individual variation over time. These analyses can inform decisions about the use of a measure in NGS research; specifically, whether the measure is reliable enough to answer the particular research question of interest, or if forgoing the use of this measure is justified to instead devote efforts to developing measures explicitly designed to optimize reliability.

Importantly, the goal of GT is to characterize the *reliability* of a measurement tool, *not* the validity. That is, while GT can determine how consistently a measurement tool is capturing a certain skill, it cannot determine if the skill being captured is ultimately relevant to the outcome or effect of interest. For example, a child who is nonverbal may receive a low score on an anxiety screener that primarily measures symptoms of anxiety that are expressed verbally. This low score may not accurately reflect the child’s anxiety level but instead the child’s inability to express anxiety verbally. GT is unable to “diagnose” and “fix” this underlying cause of decreased validity, though the issue may be reflected in GT analyses of the measure. For example, a GT analysis might indicate that person-level differences account for only a small proportion of variance in scores among children who are nonverbal; however, it is up to the researcher running the GT analysis to identify verbal ability as a potentially relevant factor, and to structure their GT analysis in a way that can address this question. Current efforts are underway to address these issues of validity specifically for measures of early development in NGS (Kelleher et al., under review). Nevertheless, understanding the reliability of measurement tools is an important step in better understanding the validity of those tools. Thus, while using GT to characterize the reliability of measurement tools does not solve all the measurement challenges that the field of NGS research faces, it does advance the field by providing a mechanism to better understand how well the existing and commonly-used measurement tools are functioning in these unique populations.

In this study, we demonstrate the advantages of using GT in NGS research by evaluating the reliability of the CSBS-ITC in three distinct NGS groups, each with a unique phenotypic profile that demonstrates the heterogeneity often observed among NGS groups. We compare the reliability of the CSBS-ITC in these NGS groups to that which is observed in a low-risk control group, providing an anchor for what might be generally considered acceptable in non-syndromic populations. Together, this work aims to provide a model for using GT to critically evaluate measurement tools used in NGS research in order to better align our study designs with the strengths and limitations of the available measurement tools in the field.

## Methods

### Participants

**Fig. 1:**
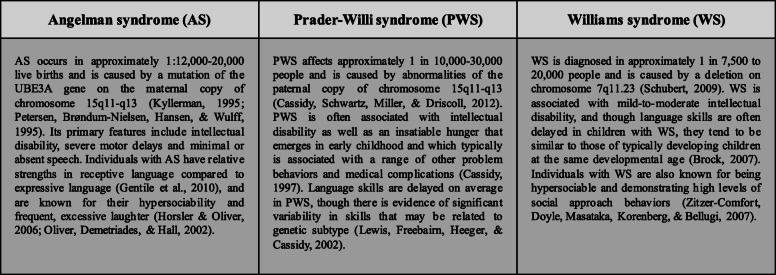
Phenotypic features of NGS groups

Participants included 172 children enrolled in the Purdue Early Phenotype Study, a longitudinal survey examining the early development of children with NGS. The present study includes children who have a diagnosis of Angelman syndrome (AS; *n* = 41), Prader-Willi syndrome (PWS; *n* = 30), or Williams syndrome (WS; *n* = 42). We also included a group of children with no genetic diagnosis (low-risk controls or LRC; *n* = 59) to provide a comparison of the expected profiles and trajectories of early social communication development in typical development. While we expect that most children with a NGS will demonstrate atypical social communication development, the three NGS groups included in this study have unique phenotypes that will allow us to examine the reliability of the CSBS-ITC across a range of phenotypic profiles that vary in the severity and variability of intellectual disability and language skills. Figure 1 includes key phenotypic features of the NGS groups included in this study.

Participants’ mothers completed surveys at 6- or 12-month intervals depending on child age, for a total of 454 measurement points across participants. Across groups and measurement points, children’s ages ranged from 1 to 60 months (*M* = 28.12, *SD* = 13.95). Table 1 includes demographic information across groups, as well as a breakdown of number of measurement points per participant by group. Cross-group analyses of CSBS-ITC scores have been previously published using a subset of the sample included in this report [15].

**Table 1:** Demographic information

Chronological Age (months)^**a**^	LRC (***n*** = 59)	AS (***n*** = 41)	PWS (***n*** = 30)	WS (***n*** = 42)
Time 1	19.15 (13.71) *n* = 59	26.18 (13.45) *n* = 41	16.38 (9.37) *n* = 30	27.06 (13.62) *n* = 42
Time 2	25.51 (14.25) *n* = 54	31.69 (13.47) *n* = 29	23.84 (10.30) *n* = 25	31.91 (12.36) *n* = 27
Time 3	31.02 (13.08) *n* = 44	29.77 (9.44) *n* = 15	28.16 (8.95) *n* = 18	38.07 (11.50) *n* = 17
Time 4	40.10 (10.57) *n* = 28	39.62 (4.13) *n* = 6	39.33 (7.35) *n* = 12	44.06 (7.91) *n* = 6
Time 5	--	--	48.62; *n* = 1	--
Attrition^b^	*n* = 10 (17%)	*n* = 8 (20%)	*n* = 4 (13%)	*n* = 10 (24%)
**Number of Observations**^**c**^	*n*	*n*	*n*	*n*
1	5	12	5	15
2	10	14	7	10
3	16	9	6	11
4	28	6	11	6
5	0	0	1	0
Total	185	91	86	92
**Demographics**	*n* (%)	*n* (%)	*n* (%)	*n* (%)
**Female**^**d**^	23 (39%)	21 (51%)	17 (57%)	21 (50%)
**Race**^**e**^				
White	54 (92%)	34 (83%)	26 (87%)	34 (81%)
Black	0 (0%)	0 (0%)	0 (0%)	1 (2%)
Native Hawaiian/Pacific Islander	0 (0%)	0 (0%)	0 (0%)	1 (2%)
Multiracial	0 (0%)	2 (5%)	1 (3%)	1 (2%)
Not Reported	5 (8%)	5 (12%)	3 (10%)	5 (12%)
**Ethnicity**				
Hispanic/Latino	1 (2%)	1 (2%)	1 (3%)	3 (7%)
Not Hispanic/Latino	53 (90%)	36 (88%)	26 (87%)	34 (81%)
Not Reported	5 (8%)	4 (10%)	3 (10%)	5 (12%)
**Child adaptive functioning** **(at second timepoint)**^**f**^	*M* (SD); *n*	*M* (SD); *n*	*M* (SD); *n*	*M* (SD); *n*
VL-3 ABC	95.22 (13.85); *n* = 41	50.14 (8.71); *n* = 21	70.81 (10.94); *n* = 21	70.36 (13.38); *n* = 25
**Household income**^**g**^ **(most recent timepoint)**	*n* (%)	*n* (%)	*n* (%)	*n* (%)
$0–$15,000	0 (0%)	1 (2%)	0 (0%)	1 (2%)
$15,001–$35,000	2 (3%)	3 (7%)	2 (7%)	6 (14%)
$35,001–$75,000	18 (31%)	9 (22%)	7 (23%)	10 (24%)
$75,001–$150,000	27 (46%)	17 (42%)	13 (43%)	18 (43%)
Over $150,000	11 (19%)	7 (17%)	5 (17%)	6 (14%)
Not reported	1 (2%)	4 (10%)	3 (10%)	1 (2%)
**Maternal Education Level**^**h**^**(most recent timepoint)**	*n* (%)	*n* (%)	*n* (%)	*n* (%)
Less than high school	1 (2%)	6 (15%)	0 (0%)	0 (0%)
High school degree	5 (8%)	8 (20%)	4 (13%)	7 (17%)
Associates degree	2 (3%)	3 (7%)	2 (7%)	4 (10%)
Some college	21 (36%)	8 (20%)	15 (50%)	15 (36%)
Bachelor’s degree	21 (36%)	16 (39%)	6 (20%)	13 (31%)
More than bachelor’s degree	8 (14%)	0 (0%)	3 (10%)	3 (7%)
Not reported	1 (2%)	0 (0%)	0 (0%)	0 (0%)

*Note*. ^a^Participants in the AS and WS groups were significantly younger than participants in the PWS and LRC groups at the first observation, *F*(3,168) = 6.42, *p* < .001

^b^Sample size decreases across measurement points reflect a combination of attrition, the ongoing nature of our study, and our flexible recruitment approach, which allows participants to enter the study at any age < 60 months, resulting in varying number of completed measurement points across participants at the time of data analysis

^c^Participants with only one observation (as opposed to repeated observations) were more likely to be in the AS or WS groups (AS *n* = 12 [29%], PWS *n* = 5 [17%], WS *n* = 15 [36%], LRC *n* = 5 [8%]). Those who completed only one observation did not differ in sex, *χ*^2^(1, *n* = 172) < .001, *p* = 1.00; average family income, *F*(1,161) = 0.051, *p* = .821; or maternal education, *F*(1,169) = 0.312, *p* = .577

^d^Groups did not differ in sex, *χ*^2^(3, *n* = 172) = 3.06, *p* = .383

^e^No participants were identified as Asian

^f^Because our VL-3 data does not include participants who dropped out of the study before the second timepoint, our estimates of adaptive functioning for each group may be biased against participants who dropped out of the study early

^g^Groups did not differ in average family income, *F*(3,159) = 0.306, *p* = .821

^h^Groups differed in maternal education, *F*(3,167) = 4.06, *p* = .008. Tukey’s post-hoc comparisons showed that participants in the AS group had mothers with significantly less education than LRCs, *p* = .004. No other groups differed significantly in maternal education (*p*’s > .213)

Inclusion criteria required that the child’s family resided in the USA and that English was the primary language spoken in the home. LRC exclusion criteria included premature birth (< 37 weeks gestation), speech delay, or having a first-degree family member diagnosed with autism spectrum disorder. We confirmed genetic status for participants in the NGS groups by medical and genetic reports provided by the participant’s family.

### Measures

#### Communication and Symbolic Behavior Scales Developmental Profile–Infant-Toddler Checklist ([3])

The CSBS-ITC is a 24-item parent-report screening checklist that assesses skills pertaining to social responses, speech abilities, and symbolic comprehension to identify infants who may be at risk for developing social communication delays. Parents choose an option that best describes their child’s behavior related to specific social communication skills. Most items are scored on a 0–2 scale (19 items; 0 = not yet, 1 = sometimes, 2 = often). Five items are scored on scales with more options (1 item with scale 0–3, 4 items with scale 0–4) that allow the parent to indicate a range that best describes their child’s behavior (e.g., “About how many words does your child use meaningfully that you recognize?” where 0 = none, 1 = 1–3, 2 = 4–10, 3 = 11–30, 4 = Over 30). All item scores are summed to create a total raw score, which ranges from 0 to 57. While the normed age range for the CSBS-ITC is from ages 6 to 24 months, the CSBS-ITC can be used in children older than 24 months who have language delays (such as those with neurogenetic syndromes), particularly when using raw scores instead of norm-referenced scores [15]. Parents completed the CSBS-ITC 1 to 5 times per child, starting when the child’s mother contacted the Neurodevelopmental Family Lab and continuing at approximately 6- or 12-month intervals, depending on the child’s age. Reliability of the CSBS-ITC total raw score in past studies is *α* = .93 for a single fixed assessment [3]. In the present study, Cronbach’s alpha was .96 for the CSBS-ITC total raw score of each participant’s first measurement point in the overall sample (including NGS and LRC together). When calculated for NGS and LRC groups individually, Cronbach’s alpha was .97 in LRCs, .90 in AS, .95 in PWS, and .95 in WS.^1^

#### Vineland Adaptive Behavior Scales–Third Edition ([16])

The VL-3 is a semi-structured parent-report interview assessing adaptive skills for individuals aged birth through 89 years. Studies have applied previous versions of the VL-3 to neurogenetic syndrome populations, including in clinical trials [17]. In this study, we used the Adaptive Behavior Composite (ABC) score to characterize adaptive functioning level for a subset of participants whose mothers had completed a follow-up phone interview (LRC *n* = 42, 71%; AS *n* = 30, 73%; PWS *n* = 21, 70%; WS *n* = 26, 62%).

### Procedure

All procedures were approved by the university’s Institutional Review Board, and caretakers provided consent for participation. We recruited families primarily through postings on social media pages (i.e., Facebook and a lab webpage), as well as a subset of WS and AS participants through the Williams Syndrome Registry and Angelman Syndrome Foundation, respectively. We recruited LRC participants through advertisements on Facebook that targeted a nationally-representative sample of mothers with young children. Mothers interested in participating in the study contacted the lab and completed screening questions to determine eligibility. After confirming eligibility, we sent mothers the link to a password-protected online survey to complete the first measurement point of the study, which included the CSBS-ITC. Due to the likelihood of developmental delays in the NGS group, we administered the CSBS-ITC to all age ranges to capture older participants whose developmental level matched the level of social communication skills included on the CSBS-ITC. After mothers completed their child’s second measurement point, we invited them to participate in a phone interview about their child, which included the VL-3.

### Data cleaning

We created an “age bin” variable as a categorical variable that indicated the developmental sequence of the participant’s measurement points. This transformation ensured that age was partially crossed with individuals instead of nested within person. Age bins ranged from 0 to 60 months at 3-month intervals, for a total of 20 age bins. We chose this interval because 3 months is a small enough age range to be fairly certain that children will make meaningful advances in skills and is also consistent with typical age groupings commonly used in many studies in the field (e.g., [18]). Each individual had measurement points assigned to 1–5 different age bins. Age bin size is reported in Table S1 as supplementary material at https://osf.io/dsb68/. After running initial analyses, we re-ran our models using different sizes of age bins to determine whether the size of the age bin affected the reliability of the CSBS-ITC Total score and found that the effect of changing age bin size was minimal. These analyses are also presented as supplemental material in Table S2.

While most items on the CSBS-ITC are rated on a scale of 0 to 2, one item is scored on a scale of 0 to 3, and four are scored on a scale from 0 to 4. Because we used raw scores as the dependent variable, it was important that all items were scored on the same scale to ensure that all items were given equal weight in models. Thus, we rescaled scores from items scored on a 0 to 3 scale by dividing the item score by 1.5, and items scored on a 0 to 4 scale by dividing by 2. These transformations resulted in all items being scored on a 0 to 2 scale.

### Analytic plan

We completed statistical analyses using PROC MIXED in SAS Software Version 9.4. We used non-parametric bootstrapping to simulate 1000 iterations of each model, the results of which were compiled and used to calculate point estimates and 95% confidence intervals [19]. All models were fitted using restricted maximum likelihood.^2^ SAS code for all analyses can be found at https://osf.io/awbrn/. We ran all multi-level models for generalizability analyses based on models and procedures reported in Cranford et al. [14], which are summarized briefly here.

### GT analysis 1

The goal of the first GT analysis was to conduct a G Study and a D Study to make decisions about the appropriate application of the CSBS-ITC total raw score in each risk group. For each group, we predicted variance in item-level scores of all 24 CSBS-ITC items from the random effects of person, age, item, and the interactions of these variables, based on variance components needed to calculate Cranford et al. [14]’s reliability estimates (Eq. 1). By predicting item-level scores of all 24 items of the CSBS-ITC, we can interpret variance estimates as the proportion of variance the variable accounted for in the CSBS-ITC total raw score.

Eq. 1:

$$ {\mathrm{Score}}_{ij k}={\mathrm{Person}}_i+{\mathrm{Age}}_j+{\mathrm{Item}}_k+{\left(\mathrm{Person}\times \mathrm{Age}\right)}_{ij}+{\left(\mathrm{Person}\times \mathrm{Item}\right)}_{ik}+{\left(\mathrm{Age}\times \mathrm{Item}\right)}_{jk}+{e}_{ij k} $$

We then conducted D Studies to calculate 4 reliability estimates for each group. These reliability estimates (*R*_*1F*_, *R*_*1R*_, *R*_*KF*_, *R*_*C*_) can be used to determine how reliably the CSBS-ITC can differentiate scores from different individuals at the same or different measurement points. We used classical test theory conventions for interpreting reliability estimates (i.e., 0.00–0.10 = virtually none, 0.11–0.40 = slight, 0.41–0.60 = fair, 0.61–0.80 = moderate, 0.81–1.00 = substantial [20];). In the equations below, *m* represents the number of items (in our study, *m* = 24) and *k* represents the modal number of measurement points (in our study, *k* = 4).

*R*_*1F*_ estimates the ability of the CSBS-ITC to differentiate between similarly-aged children (i.e., children within a single age bin) (Eq. 2).

Eq. 2:

$$ {R}_{1F}=\frac{\sigma_{\mathrm{Person}}^2+\left[{\sigma}_{\mathrm{Person}\times \mathrm{Item}}^2/m\right]}{\sigma_{\mathrm{Person}}^2+\left[{\sigma}_{\mathrm{Person}\times \mathrm{Item}}^2/m\right]+\left[{\sigma}_{\mathrm{Error}}^2/m\right]} $$

*R*_*1R*_ estimates the reliability of the CSBS-ITC when differentiating children at measurement points from randomly selected age bins (Eq. 3).

Eq. 3:

$$ {R}_{1R}=\frac{\sigma_{\mathrm{Person}}^2+\left[{\sigma}_{\mathrm{Person}\times \mathrm{Item}}^2/m\right]}{\sigma_{\mathrm{Person}}^2+\left[{\sigma}_{\mathrm{Person}\times \mathrm{Item}}^2/m\right]+{\sigma}_{\mathrm{Age}}^2+{\sigma}_{\mathrm{Person}\times \mathrm{Age}}^2+\left[{\sigma}_{\mathrm{Error}}^2/(m)\right]} $$

*R*_*KF*_ estimates the reliability of the CSBS-ITC to differentiate children when averaging scores across all available measurement points for each child (Eq. 4).

Eq. 4:

$$ {R}_{KF}=\frac{\sigma_{\mathrm{Person}}^2+\left[{\sigma}_{\mathrm{Person}\times \mathrm{Item}}^2/m\right]}{\sigma_{\mathrm{Person}}^2+\left[{\sigma}_{\mathrm{Person}\times \mathrm{Item}}^2/m\right]+\left[{\sigma}_{\mathrm{Error}}^2/\left(k\times m\right)\right]} $$

*R*_*c*_ estimates the reliability of the CSBS-ITC to detect systematic variation in skills over time (Eq. 5).

Eq. 5:

$$ {R}_C=\frac{\sigma_{\mathrm{Person}\times \mathrm{Age}}^2}{\sigma_{\mathrm{Person}\times \mathrm{Age}}^2+\left[{\sigma}_{\mathrm{Error}}^2/m\right]} $$

### GT analysis 2

The goal of the second GT analysis was to conduct G Studies and D Studies for each individual CSBS-ITC subscale (i.e., social, speech, symbolic) to determine whether the reliability of the CSBS-ITC varies based on the type of skill being measured. We first estimated item-level variance for items in each composite of the CSBS-ITC (social 13 items; speech 5 items; symbolic 6 items) from the random effects of person, age, item content, and the interactions of these variables (Eq. 1). We then calculated reliability estimates for each composite by risk group (Eq. 2, 3,4,5) using the number of items within each subscale as the value for index *m* (i.e., social *m* = 13; speech *m* = 5; symbolic *m* = 6).

## Results

### Generalizability analyses

#### GT analysis 1: CSBS-ITC total raw score by risk status

**Fig. 2:**
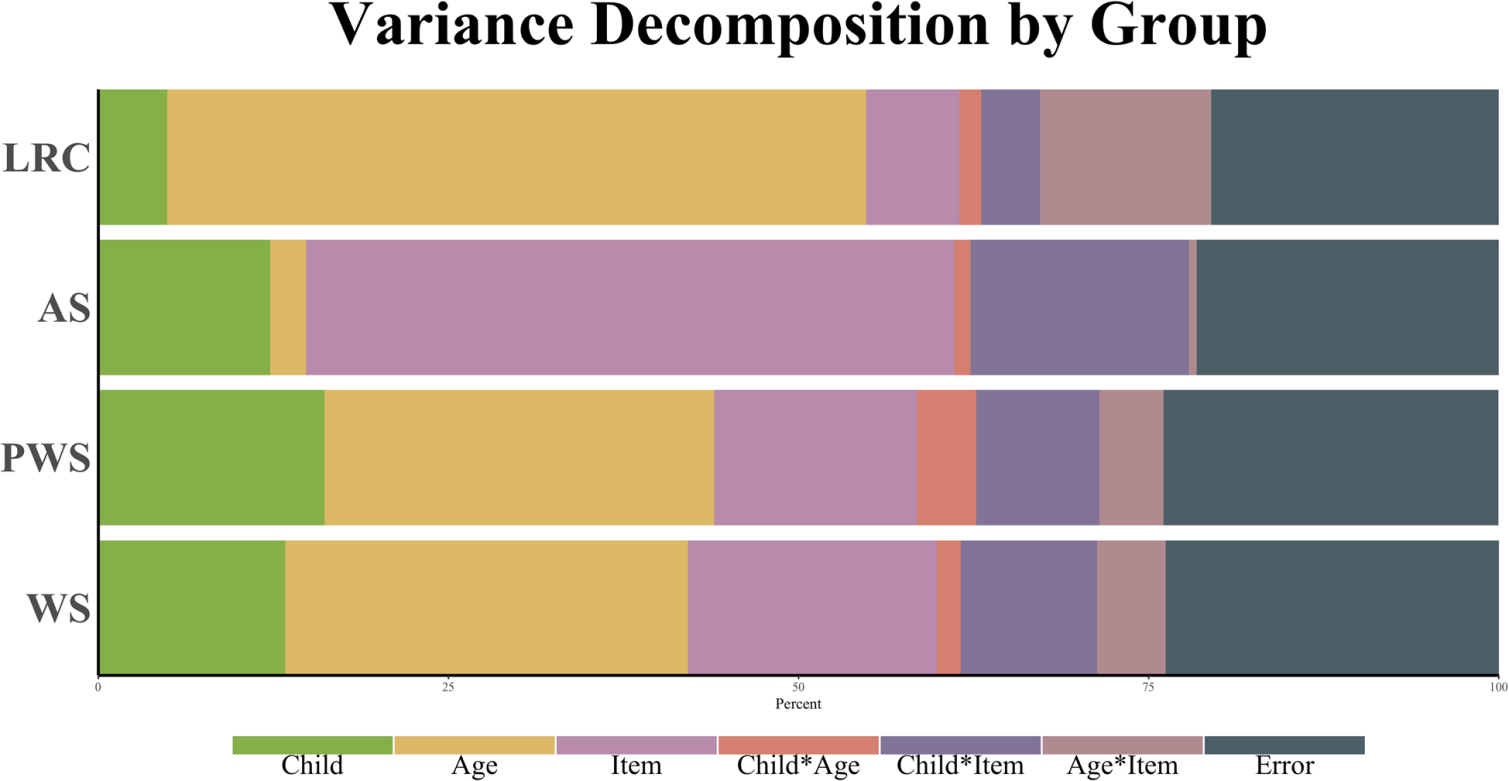
Visual representation of percent variance in CSBS-ITC composite raw scores by risk status

We first conducted a G Study and a D Study to evaluate the reliability of the CSBS-ITC total raw score for each risk group separately. Variance decomposition of the CSBS-ITC total raw score from the G Study is reported in Table 2 and Fig. 2. Results of the G Studies suggested that groups differed in terms of which factors contributed the most variance in CSBS-ITC total raw scores. In the LRC group, age accounted for 50% of the variance and the age-by-item interaction accounted for 12% of the variance in CSBS-ITC total raw scores. Person (5%), item (7%), and the person-by-item interaction (4%) accounted for relatively less variance in CSBS-ITC total raw scores in the LRC group, and very little variance (< 2%) was accounted for the by person-by-age interaction.

**Table 2:** Variance decomposition of CSBS-ITC item-level raw scores from second G Study by risk status

	LRC		AS		PWS		WS	
Source of variance	Variance	Percentage	Variance	Percentage	Variance	Percentage	Variance	Percentage
σ^2^_Person_	0.027	4.86%	0.083	12.51%	0.117	16.27%	0.096	13.44%
σ^2^_Age_	0.276	49.61%	0.016	2.43%	0.195	27.13%	0.199	27.91%
σ^2^_Item_	0.038	6.86%	0.308	46.60%	0.106	14.74%	0.128	17.97%
σ^2^_Person*Age_	0.009	1.58%	0.007	1.11%	0.031	4.29%	0.013	1.80%
σ^2^_Person*Item_	0.024	4.30%	0.101	15.35%	0.064	8.91%	0.073	10.18%
σ^2^_Age*Item_	0.068	12.14%	0.004	0.63%	0.033	4.63%	0.034	4.71%
σ^2^_Residual_	0.115	20.65%	0.141	21.37%	0.173	24.02%	0.171	23.99%
**Total**	**0.557**	**100%**	**0.661**	**100%**	**0.719**	**100%**	**0.715**	**100%**

In contrast, variance decompositions for the NGS groups tended to show different patterns than the LRC group. Age accounted for much lower proportions of variance in the NGS groups compared to the LRC group, and while age still accounted for the highest proportion of variance within the PWS (27%) and WS (28%) groups, it accounted for very little variance in the AS group (2%). Consequently, person- and item-level factors accounted for much more variance in NGS CSBS-ITC total raw scores. In AS, the majority of variance in scores was due to item (47%) and the person-by-item interaction (15%). These components similarly accounted for relatively high proportions of variance in the WS group (item = 18%; person-by-item = 10%), though person-level factors also contributed a relatively high proportion of variance (13%). In the PWS group, person accounted for the next highest proportion of variance (16%) after age, followed by item (15%) and the person-by-item interaction (9%). Thus, while age appeared to account for large portions of variance across most groups, item- and person-level factors were also important to the variance decomposition of CSBS-ITC total raw scores in the NGS groups, and their relative contributions tended to differ slightly among the NGS groups.

Point estimates and confidence intervals for the reliability coefficients of CSBS-ITC total raw score by group are reported in Table 3. In the LRC group, the reliability of the CSBS-ITC total raw score was substantial at a single fixed measurement point and when averaged across all measurement points (*R*_*1F*_ = .85 and *R*_*KF*_ = .96, respectively). The CSBS-ITC total raw score had moderate reliability to detect systematic change over time among LRC participants (*R*_*C*_ = .64) but was not reliable when taken from a single random measurement point (*R*_*1R*_ = .09). Patterns of reliability in the NGS groups were generally similar to the LRC group. *R*_*1F*_ and *R*_*KF*_ were substantial across all NGS groups. The ability of the CSBS-ITC total raw score to detect systematic change over time was substantial in the PWS group (*R*_*C*_ = .81), moderate in the WS group (*R*_*C*_ = .63), and fair in the AS group (*R*_*C*_ = .53). Its ability to differentiate children with AS at any single random measurement point was moderate (*R*_*1R*_ = .75) but was only fair in the PWS and WS groups. Thus, it appeared that for all groups, the CSBS-ITC total raw score was most reliable when it was averaged across all available measurement points. It was least reliable when comparing scores from randomly selected measurement points for all groups except the AS group, for which it was least reliable when detecting systematic change in scores over time.

**Table 3:** Reliability coefficients of CSBS-ITC total raw score by risk status

Reliability coefficient	LRC	AS	PWS	WS	Interpretation
*R*_*1F*_	0.85 [0.83–0.87]	0.94 [0.92–0.95]	0.94 [0.94–0.95]	0.93 [0.92–0.94]	Ability of the CSBS-ITC total taw score to differentiate children at a single fixed measurement point.
*R*_*1R*_	0.09 [0.08–0.10]	0.75 [0.67–0.82]	0.34 [0.30–0.38]	0.31 [0.28–0.35]	Ability of the CSBS-ITC total raw score to differentiate children when measured at single random measurement point.
*R*_*KF*_	0.96 [0.95–0.96]	0.98 [0.98–0.99]	0.99 [0.98–0.99]	0.98 [0.98–0.98]	Reliability of average CSBS-ITC total raw score across all measurement points.
*R*_*C*_	0.64 [0.55–0.72]	0.53 [0.28–0.69]	0.81 [0.75–0.85]	0.63 [0.47–0.74]	Reliability of systematic change in CSBS-ITC total raw score from one measurement point to another.

*Note*. 95% confidence intervals are included in brackets below each reliability estimate

#### GT analysis 2: CSBS-ITC composite raw scores by risk status

**Fig. 3:**
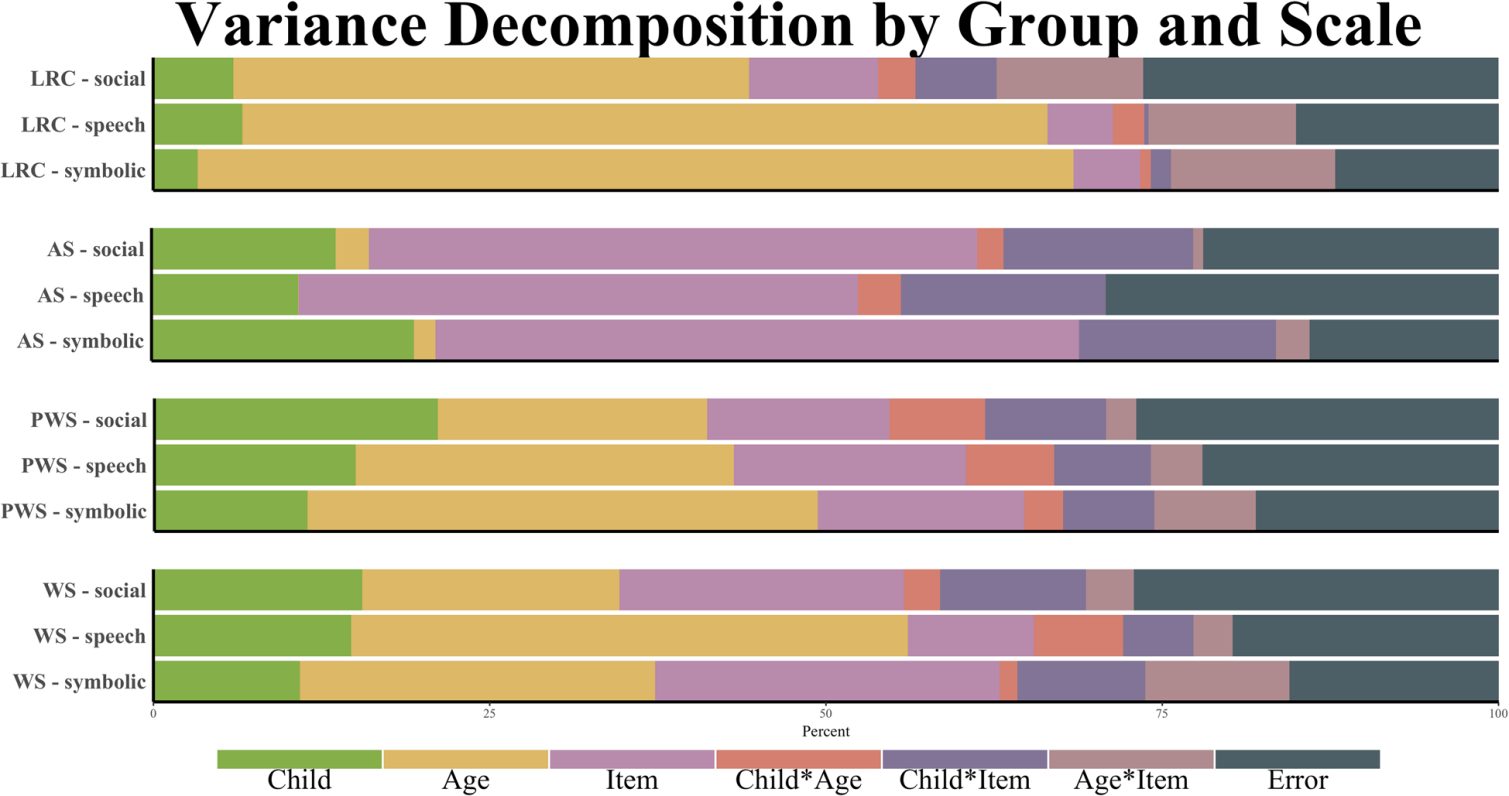
Visual representation of percent variance in CSBS-ITC composite raw scores by risk status

Given that CSBS-ITC items are divided into three composites that target related but distinct developmental domains (social, speech, and symbolic skills), we conducted a second set of GT analyses for these subsets of items to determine how reliability differed by CSBS-ITC composite in each group. Variance decomposition by composite and risk status are reported in Table 4 and Fig. 3. In the LRC group, variance accounted for by age varied the most across composites, accounting for the highest proportion in the symbolic composite (64%) and a much lower proportion in the social composite (38%). All other factors accounted for similar proportions of variance across composites (all within 6% across composites). This fluctuation in variance due to age was observed in the PWS and WS groups as well. In the PWS group, similar to the LRC group, age accounted for the highest proportion of variance in the symbolic composite (36%) and the least proportion of variance in the social composite (19%). However, in the WS group, age accounted for the most variance in the speech composite (39%) and the least variance in the social composite (18%). In contrast, in the AS group, age accounted for a similarly low proportion of variance across all composites (range 0–2%). In fact, all variance components accounted for relatively similar proportions of variance across composites in the AS group, with person-level factors showing this highest fluctuation—person-level factors accounted for 20% variance in symbolic composite scores, while only accounting for 10% variance in speech composite scores (all other fluctuations between composites in AS were < 8%). Variance due to person-level factors fluctuated by 10% in the PWS group (accounted for 11% variance in symbolic composite scores, 21% variance in social composite scores), but by less than 5% in the WS group. However, variance due to item content varied considerably in the WS group, accounting for 27% variance in symbolic composite scores but only 10% variance in speech composite scores. Variance estimates for item content varied by less than 4% across composites in the PWS group. Overall, variation accounted for by age appeared to vary the most based on the particular skill domain in question and also showed nuanced fluctuation among the composites for each group. Variance due to person-level factors was also relatively affected by composite for the NGS groups, as was variance due to item-level factors (despite the fact that items within composites should be more similar to each other than to those from the other composites).

**Table 4:** Variance decomposition of CSBS-ITC item-level scores by risk status and composite

	LRC		AS		PWS		WS	
Source of Variance	Variance	Percentage	Variance	Percentage	Variance	Percentage	Variance	Percentage
**Social**								
σ^2^_Person_	0.031	5.93%	0.105	13.78%	0.146	21.28%	0.111	15.77%
σ^2^_Age_	0.198	37.99%	0.018	2.37%	0.132	19.18%	0.127	18.09%
σ^2^_Item_	0.051	9.78%	0.345	45.40%	0.093	13.56%	0.149	21.29%
σ^2^_Person*Age_	0.015	2.84%	0.015	1.98%	0.051	7.39%	0.020	2.84%
σ^2^_Person*Item_	0.033	6.25%	0.106	13.94%	0.064	9.36%	0.080	11.38%
σ^2^_Age*Item_	0.055	10.53%	0.006	0.84%	0.015	2.25%	0.025	3.54%
σ^2^_Residual_	0.139	26.67%	0.165	21.68%	0.185	26.99%	0.191	27.19%
**Total variance**	0.521	100.00%	0.760	100.00%	0.686	100.00%	0.703	100.00%
**Speech**								
σ^2^_Person_	0.041	6.54%	0.044	10.39%	0.112	15.44%	0.107	14.95%
σ^2^_Age_	0.373	59.28%	0.002	0.39%	0.196	27.11%	0.279	38.78%
σ^2^_Item_	0.032	5.07%	0.173	41.10%	0.127	17.59%	0.073	10.22%
σ^2^_Person*Age_	0.016	2.53%	0.013	3.19%	0.048	6.59%	0.053	7.31%
σ^2^_Person*Item_	0.004	0.62%	0.066	15.65%	0.050	6.86%	0.039	5.41%
σ^2^_Age*Item_	0.070	11.12%	0.001	0.27%	0.030	4.10%	0.022	3.03%
σ^2^_Residual_	0.094	14.85%	0.122	29.01%	0.162	22.31%	0.146	20.29%
Total variance	0.629	100.00%	0.420	100.00%	0.724	100.00%	0.719	100.00%
**Symbolic**								
σ^2^_Person_	0.018	3.23%	0.120	19.68%	0.077	11.20%	0.074	11.01%
σ^2^_Age_	0.358	64.49%	0.010	1.61%	0.247	36.08%	0.164	24.33%
σ^2^_Item_	0.029	5.18%	0.298	48.76%	0.114	16.59%	0.180	26.74%
σ^2^_Person*Age_	0.005	0.90%	0.001	0.21%	0.022	3.21%	0.012	1.72%
σ^2^_Person*Item_	0.008	1.53%	0.084	13.72%	0.046	6.70%	0.066	9.77%
σ^2^_Age*Item_	0.069	12.42%	0.014	2.34%	0.054	7.93%	0.068	10.08%
σ^2^_Residual_	0.068	12.25%	0.084	13.67%	0.125	18.29%	0.110	16.34%
Total variance	0.556	100.00%	0.612	100.00%	0.685	100.00%	0.672	100.00%

Point estimates and confidence intervals for reliability estimates of each composite by group are reported in Table 5. For the social composite in the LRC group, reliability estimates were poor for *R*_*1R*_ and fair for *R*_*C*_, but were moderate or better for *R*_*1F*_ and *R*_*KF*_. In all three NGS groups, the social composite had substantial *R*_*1F*_ and *R*_*KF*_. *R*_*C*_ was moderate in the PWS group but was fair in the AS and WS groups. In contrast, *R*_*1R*_ for the social composite was fair in the PWS and WS groups, but moderate in the AS group. For the speech composite, *R*_*KF*_ was substantial, *R*_*1F*_ was moderate, *R*_*C*_ was fair, and *R*_*1F*_ was not reliable in the LRC group. In the NGS groups, *R*_*1F*_, *R*_*KF*_, and *R*_*C*_ were all moderate or better for all composites except for *R*_*C*_ in AS. Finally, for the symbolic composite in the LRC group, *R*_*1F*_ and *R*_*KF*_ were moderate or better, but *R*_*1R*_ and *R*_*C*_ were slight or worse. For the symbolic composite in the NGS groups, *R*_*1F*_ and *R*_*KF*_ were moderate or better for all groups, as was *R*_*1R*_ in the AS group. *R*_*C*_ was fair or worse in all three NGS groups. *R*_*1R*_ was slight for the symbolic composite in both the PWS and WS groups. Thus, overall, the ability of the CSBS-ITC composites to differentiate children at fixed measurement points or by averaging scores across measurement points was generally moderate or better across composites and groups, whereas the ability to differentiate children at randomly selected measurement points was fair or worse across all composites for all groups except the AS group. The ability of the CSBS-ITC composites to detect change was the most variable across groups and composites, tending to be highest for the social and speech composites and for the PWS and WS groups.

**Table 5:** Reliability coefficients of CSBS-ITC composite raw scores by risk status

	LRC	AS	PWS	WS
**Social**				
*R*_*1F*_	0.76 [0.71–0.79]	0.90 [0.87–0.92]	0.91 [0.90–0.93]	0.89 [0.86–0.91]
*R*_*1R*_	0.13 [0.11–0.16]	0.71 [0.62–0.80]	0.43 [0.37–0.50]	0.42 [0.35–0.50]
*R*_*KF*_	0.93 [0.91–0.94]	0.97 [0.96–0.98]	0.98 [0.97–0.98]	0.97 [0.96–0.98]
*R*_*C*_	0.57 [0.45–0.66]	0.51 [0.23–0.68]	0.78 [0.70–0.83]	0.55 [0.32–0.70]
**Speech**				
*R*_*1F*_	0.69 [0.62–0.75]	0.69 [0.55–0.81]	0.79 [0.72–0.84]	0.79 [0.73–0.86]
*R*_*1R*_	0.09 [0.07–0.12]	0.59 [0.40–0.74]	0.31 [0.24–0.38]	0.24 [0.18–0.30]
*R*_*KF*_	0.90 [0.87–0.92]	0.90 [0.83–0.94]	0.94 [0.91–0.96]	0.94 [0.91–0.96]
*R*_*C*_	0.43 [0.12–0.63]	0.32 [0.00–0.58]	0.58 [0.36–0.74]	0.62 [0.39–0.78]
**Symbolic**				
*R*_*1F*_	0.62 [0.52–0.71]	0.90 [0.87–0.93]	0.80 [0.71–0.86]	0.82 [0.76–0.87]
*R*_*1R*_	0.05 [0.03–0.07]	0.84 [0.71–0.92]	0.23 [0.15–0.31]	0.31 [0.23–0.40]
*R*_*KF*_	0.87 [0.81–0.91]	0.97 [0.96–0.98]	0.94 [0.91–0.96]	0.95 [0.93–0.96]
*R*_*C*_	0.28 [0.00–0.52]	0.06 [0.00–0.38]	0.48 [0.15–0.69]	0.33 [0.00–0.64]

*Note*. 95% confidence intervals are included in brackets below each reliability estimate. See Table 3 for brief descriptions of interpretation for reliability coefficients.

## Discussion

Characterizing early development in children with severe developmental delays is challenging for a number of reasons, including the limited repertoire of developmental tools appropriate to these populations, as well as the unclear psychometric rigor of existing measures. This study used Generalizability theory to evaluate the reliability of a popular early social communication screening measure, the CSBS-ITC, when used in NGS populations. We present three major findings. First, for all groups, reliability of the CSBS-ITC was best when averaging across all measurement points and was generally similar or better in the NGS groups compared to the LRC group. Second, the patterns of variance decomposition for the CSBS-ITC for NGS groups varied from patterns observed in the LRC group, particularly related to the amount of variance due to age, person, and item. Third, the CSBS-ITC performed differentially across the NGS groups in terms of reliability and variance decomposition, suggesting the CSBS-ITC may capture nuanced phenotypic features of the three NGS groups. Overall, despite differences in variance decomposition across groups, it appears that the CSBS-ITC has similar or better reliability in NGS compared to low-risk controls.

The CSBS-ITC generally demonstrated similar or better reliability in the NGS groups as it did in the LRC group. This suggests that the CSBS-ITC will be similarly reliable at detecting levels and changes in social communication skills in NGS populations as it is in LRC. The CSBS-ITC was substantially reliable when it was used among children with similar age ranges and when averaged across all available measurement points. The ability of the CSBS-ITC to detect systematic change over time was quite variable across the different groups and composites. It tended to be most reliable in the PWS group, with moderate or substantial *R*_*C*_ reliability for the total and composite scores. This suggests that variation in CSBS-ITC scores over time reflected reliable within-person change in the PWS group, and not change due to error. This was also true of the LRC and WS groups for the CSBS-ITC total score and for the WS group with the speech composite. In all other cases, though, *R*_*C*_ reliability was fair or worse, particularly when using a single composite instead of the total score. This suggests that variation in these CSBS-ITC score trajectories reflected a large amount of error. Importantly, it does *not* imply that the average trajectory of change across individuals was low; it suggests that *differences* in trajectories were likely due to error. Thus, studies that are using the CSBS-ITC to measure change over time and are interested in the heterogeneity of social communication skill trajectories would be best advised to use the total score instead of focusing on a single composite.

The reliability of CSBS-ITC scores from randomly selected measurement points generally presented the worst reliability estimates across groups. This likely reflected the developmental nature of the CSBS-ITC. That is, because children are continuously developing new skills, it is not reasonable to assume their score from any random measurement point will reflect their overall ability, making it necessary to know the ages at which the scores were obtained in order to meaningfully compare two CSBS-ITC scores. Statistically adjusting for age in predictive models is one way to mitigate this problem, but even then our findings suggest that it could be problematic to compare the CSBS-ITC scores of two children whose ages are more than 3 months apart because it would not take into account the other sources of systematic variation. Thus, using the CSBS-ITC to differentiate between children with skills reported at randomly selected measurement points would not be recommended for any population. This study model is sometimes used in research with children with NGS due to the challenges of recruiting children with these rare syndromes, especially at very young ages, including our own work [15]. Our findings suggest that this approach may not be appropriate for future work using the CSBS-ITC, and—more broadly—that GT may help researchers make such determinations when deciding how to analyze NGS data on a case-by-case basis.

The AS group tended to demonstrate a different pattern of CSBS-ITC reliability than the other NGS groups, such that the ability to differentiate children from randomly selected measurement points was better than in the other NGS groups, and the ability to detect systematic change was lowest in the AS group. The higher *R*_*1R*_ suggests that children of varying ages can still be compared reliably within the AS group. Furthermore, the low *R*_*C*_ in the AS group suggests that variation in CSBS-ITC scores likely reflected mostly error within the age range included in our study. This is consistent with the AS phenotype, where communication skills are often delayed beyond what might even be expected for other NGS groups and change more slowly over time. Our findings suggest that this phenotypic feature may be an obstacle to the reliability of the CSBS-ITC when used across time in AS.

With regard to variance decomposition, we found that age tended to contribute large proportions of variance across all groups except AS. Age accounted for the largest proportion of variance in the LRC group, whereas variance was more evenly distributed among other components in the PWS and WS groups. This finding suggests that in the LRC group, children are developing systematically as a function of their age with very little variation across individuals. That is, their development is relatively homogenous. This finding is consistent with our expectation that children with no known risk for developmental delay will acquire early social communication skills along very similar trajectories, regardless of other factors that may be specific to the child. This was not the case in the NGS groups. Here, we saw that individual factors and the type of early social communication skill being measured were similarly important in determining a child’s score on any given item on the CSBS-ITC. Again, this finding is consistent with our understanding that early development in NGS is highly heterogeneous, thus it is unsurprising that factors other than developmental timelines—such as genetic status or other medical diagnoses—are going to more strongly influence the child’s development of early social communication skills.

The fact that age accounted for such a small proportion of variance in CSBS-ITC scores in the AS group—who generally exhibit the greatest socio-communicative delays relative to the NGS groups—suggests that children in this group did not demonstrate much consistent developmental variability in their performance of these early social communication skills. Instead, item accounted for the highest proportion of variance, indicating that children with AS tended to consistently score higher or lower on certain items of the CSBS-ITC and varied minimally in their scores over time. This may suggest that for children with AS, it is not necessary to collect multiple reports of the CSBS-ITC across the age range reflected in our study, as scores may not be expected to change much with time. Notably, it is possible that age may account for more variability in CSBS-ITC scores for children older than those included in our study. However, overall, the differences in variance decomposition across the four groups demonstrated that different factors contributed to variation in CSBS-ITC scores differentially, both between populations that are and are not at risk for developmental delays and among three high-risk populations with different phenotypic profiles.

Overall, we were able to use GT to better understand what factors contribute most to variance in CSBS-ITC scores and to compare the reliability of the CSBS-ITC in NGS populations with that of the populations in which it is most commonly used. In this way, we were better able to understand the utility of this measure and whether it can be used appropriately in populations with known developmental delays. As our findings show, this is often a nuanced answer. The reliability of the CSBS-ITC score varied tremendously—from virtually none to substantial reliability—depending on the way the score was being used and in which group. This highlights the importance of exploring multiple facets of reliability, which are typically not obtained using standard reliability methods but which can be estimated using GT analyses, and determining *which* facets of reliability are relevant for a particular measure in a given population.

Importantly, there are several additional applications of GT which were not possible to explore with the data used in the current study but which may nonetheless be relevant to developmental research. First, researchers may wish to include rater (e.g., mother, father, teacher, clinician, self-report) as a variance component in their generalizability analyses to determine the amount of variance due to certain respondents tending to answer items about the child in a systematic way (e.g., [21]). Second, some developmental measures have different scoring formats (e.g., basal and ceiling rules, item sets) that may warrant calculating reliability estimates by item set or for items that were directly administered compared to those above or below the ceiling and floor items. Third, GT can be used to optimize reliability for certain measures. For example, behavioral assessments are common in developmental research but can be heavily influenced by the child’s behavior on any given day. Using GT, researchers can determine how many measurement points are required to result in stable and substantial reliability of the measure they choose to assess the skill of interest (e.g., [22]). These additional applications can further assist in evaluating and improving the utility of common measures in NGS populations.

There are several limitations to this study. First, our sample was demographically homogeneous, preventing us from exploring the effects of race and ethnicity on our generalizability analyses. Second, while inclusion criteria required English be the primary language spoken in the home, we did not evaluate the multilingual status of children in the present study. Given the CSBS-ITC’s focus on communication and language skills, particularly the speech composite, we were unable to evaluate this potentially important facet of variance. Third, due to the web-based nature of this study, we were unable to examine how the CSBS-ITC scores were related to validation measures, such as developmental or language testing, which are typically collected through in-person assessment. While these comparisons were reported in the initial CSBS-ITC development study, they have yet to be explored in populations with severe developmental delays and should therefore be included in future studies. Finally, while the three NGS groups chosen for this study represent a wide range of developmental profiles, it is likely that these findings may not generalize to other NGS populations with different phenotypes.

## Conclusions

GT provides a method for better understanding measurement variance and score reliability and is particularly useful for research with NGS populations for which there are often a lack of well-validated measures that can be used to characterize early development. We found that CSBS-ITC item score variance tended to be differentially accounted for by factors such as child age, individual differences, and item content across the NGS and LRC groups. However, CSBS-ITC reliability tended to be as good or better in the NGS groups compared to the LRC group, suggesting the CSBS-ITC can generally be used to reliably measure early social communication levels and trajectories in NGS populations. Thus, GT is a useful tool for quantifying the reliability of both new and existing measures in NGS populations, particularly with regard to reliability across periods of change.

## Supplementary information

**Additional file 1: Table S1.** Age Bin Cell Size by Group.

**Additional file 2.** Age Bin Comparisons.

## Data Availability

The datasets generated and/or analyzed during the current study are available in the OSF repository, [https://osf.io/dsb68/?view_only=3c65367a3a814226b7a2c8ed422064ae].

## Abbreviations

NGS: Neurogenetic syndrome
GT: Generalizability theory
CSBS-ITC: Communication and Symbolic Behavior Scales–Infant-Toddler Checklist
TD: Typically developing
G Study: Generalizability study
D Study: Decision study
ANOVA: Analysis of variance
AS: Angelman syndrome
PWS: Prader-Willi syndrome
WS: Williams syndrome
LRC: Low-risk control
VL-3: Vineland Adaptive Behavior Scales, Third Edition
ABC: Adaptive Behavior Composite

## Footnotes

1 Cronbach’s alpha should approximate *R*_*1F*_ estimates. Our Cronbach’s alpha estimates for the first measurement point are generally higher than our *R*_*1F*_ estimates (which use all available measurement points), suggesting that the first measurement point may be the most reliable for our groups except for AS.

2 We used the “nlminb” optimizer and set optCtrl = list(maxfun = 2e5), as several models did not converge when using the default optimizer. The model predicting speech composite scores in AS converged using these settings but was not positive definite, as the age*item variance component was estimated to be 0.
