# Supplementary material for: Using generalizability theory to evaluate the comparative reliability of developmental measures in neurogenetic syndrome and low-risk populations
Source: J Neurodev Disord. 2020 Jun 5;12:16. doi: 10.1186/s11689-020-09318-1 (PMC7275516; doi:10.1186/s11689-020-09318-1)
Supplement: Supplementary file 1 — Additional file 1: Table S1. Age Bin Cell Size by Group. [file 11689_2020_9318_MOESM1_ESM.docx]

Table S1

*Age Bin Cell Size by Group*

|  | **Age Bin Ranges (in months)** | | | | | | | | | | | | | | | | | | | |
| --- | --- | --- | --- | --- | --- | --- | --- | --- | --- | --- | --- | --- | --- | --- | --- | --- | --- | --- | --- | --- |
|  | **0-3** | **3-6** | **6-9** | **9-12** | **12-15** | **15-18** | **18-21** | **21-24** | **24-27** | **27-30** | **30-33** | **33-36** | **36-39** | **39-42** | **42-45** | **45-48** | **48-51** | **51-54** | **54-57** | **57-60** |
| **LRC** | 5 | 7 | 14 | 10 | 13 | 11 | 10 | 10 | 12 | 17 | 14 | 10 | 11 | 6 | 8 | 7 | 6 | 5 | 5 | 4 |
| **AS** | 0 | 0 | 2 | 3 | 4 | 10 | 9 | 7 | 8 | 8 | 9 | 9 | 1 | 4 | 3 | 4 | 2 | 4 | 2 | 2 |
| **PWS** | 1 | 1 | 5 | 6 | 7 | 7 | 12 | 7 | 7 | 6 | 6 | 5 | 4 | 3 | 2 | 3 | 2 | 2 | 0 | 0 |
| **WS** | 0 | 1 | 3 | 2 | 4 | 4 | 10 | 4 | 7 | 7 | 1 | 8 | 1 | 7 | 7 | 2 | 4 | 6 | 1 | 3 |
| **Total** | **6** | **9** | **24** | **21** | **28** | **32** | **41** | **28** | **34** | **38** | **40** | **32** | **17** | **20** | **20** | **16** | **14** | **17** | **8** | **9** |
|  |  |  |  |  |  |  |  |  |  |  |  |  |  |  |  |  |  |  |  |  |
