# Supplementary material for: Using generalizability theory to evaluate the comparative reliability of developmental measures in neurogenetic syndrome and low-risk populations
Source: J Neurodev Disord. 2020 Jun 5;12:16. doi: 10.1186/s11689-020-09318-1 (PMC7275516; doi:10.1186/s11689-020-09318-1)
Supplement: Supplementary file 2 — Additional file 2. Age Bin Comparisons. [file 11689_2020_9318_MOESM2_ESM.docx]

**Age Bin Comparisons**

**Analytic Plan**

To demonstrate how the optimal interval to use for age binning may be driven by the research question and purpose of the study, we conducted analyses to demonstrate the effect of using various age bins to calculate reliability estimates. To do this, we re-ran GT Analysis 2 using 3 variations of binning for chronological age (1-month, 6-month, and 12-month bins) in addition to the 3-month bins used in the primary analyses and qualitatively compared the reliability estimates produced by each age bin.

**Results**

While primary models categorized age into 3-month bins, it is possible that reliability estimates may be further optimized by using wider or more narrow age bins, or furthermore, that the optimal age bin may vary depending on risk status. Thus, we re-ran the D Study from the second GT analysis using 3 different age bins (1-month, 6-month, 12-month; Table S2) and compared them to the original models using the 3-month age bin (Table 3). In the LRC group, reliability estimates were minimally improved using different age bins (all changes in estimates < .02), suggesting that 3-month age bins adequately capture meaningful variance in age for this group. In the NGS groups, 1-month age bins tended to yield marginally better reliability estimates than 3- 6-, and 12-month age bins, with the exception of R_C_ in the AS group which produced the highest estimate when using a 12-month age bin (improved from .56 to .60). The largest improvements from the initial models using 3-month age bins occurred for R_1R_ estimates, which remained poor for the PWS and WS group (R_1R_’s < .42) but increased from acceptable to good in the AS group (improved from .74 to .80). All other improvements in reliability estimates were minimal (< .06). Across groups, R_1R_ and R_C_ tended to be most affected by variation in age binning, with reliability estimates varying by up to .16. Overall, choice of age bin can have a minimal impact on the interpretation of the reliability of a measure.

Table S2

*Reliability Coefficients of CSBS-ITC Total Raw Scores by Risk Status and Age Bin*

|  |  | LRC | | | |  | AS | | | |  | PWS | | | |  | WS | | | |
| --- | --- | --- | --- | --- | --- | --- | --- | --- | --- | --- | --- | --- | --- | --- | --- | --- | --- | --- | --- | --- |
|  |  | 1m | 3m | 6m | 12m |  | 1m | 3m | 6m | 12m |  | 1m | 3m | 6m | 12m |  | 1m | 3m | 6m | 12m |
| R_1F_ |  | 0.85 | 0.86 | 0.86 | 0.82 |  | 0.94 | 0.93 | 0.93 | 0.93 |  | 0.94 | 0.94 | 0.94 | 0.94 |  | 0.94 | 0.93 | 0.93 | 0.94 |
| R_1R_ |  | 0.10 | 0.09 | 0.09 | 0.10 |  | 0.80 | 0.74 | 0.69 | 0.64 |  | 0.41 | 0.33 | 0.29 | 0.34 |  | 0.42 | 0.30 | 0.29 | 0.30 |
| R_KF_ |  | 0.96 | 0.96 | 0.96 | 0.95 |  | 0.98 | 0.98 | 0.98 | 0.98 |  | 0.99 | 0.99 | 0.98 | 0.98 |  | 0.98 | 0.98 | 0.98 | 0.98 |
| R_C_ |  | 0.67 | 0.65 | 0.67 | 0.55 |  | 0.51 | 0.56 | 0.55 | 0.60 |  | 0.85 | 0.81 | 0.72 | 0.69 |  | 0.69 | 0.63 | 0.62 | 0.63 |
